# Human Behavior-Based Particle Swarm Optimization

**DOI:** 10.1155/2014/194706

**Published:** 2014-04-17

**Authors:** Hao Liu, Gang Xu, Gui-yan Ding, Yu-bo Sun

**Affiliations:** ^1^School of Mathematics and Statistics, Beijing Institute of Technology, Beijing 100081, China; ^2^School of Science, University of Science and Technology Liaoning, Anshan 114051, China; ^3^Department of Mathematics, Nanchang University, Nanchang 330031, China

## Abstract

Particle swarm optimization (PSO) has attracted many researchers interested in dealing with various optimization problems, owing to its easy implementation, few tuned parameters, and acceptable performance. However, the algorithm is easy to trap in the local optima because of rapid losing of the population diversity. Therefore, improving the performance of PSO and decreasing the dependence on parameters are two important research hot points. In this paper, we present a human behavior-based PSO, which is called HPSO. There are two remarkable differences between PSO and HPSO. First, the global worst particle was introduced into the velocity equation of PSO, which is endowed with random weight which obeys the standard normal distribution; this strategy is conducive to trade off exploration and exploitation ability of PSO. Second, we eliminate the two acceleration coefficients *c*_1_ and *c*_2_ in the standard PSO (SPSO) to reduce the parameters sensitivity of solved problems. Experimental results on 28 benchmark functions, which consist of unimodal, multimodal, rotated, and shifted high-dimensional functions, demonstrate the high performance of the proposed algorithm in terms of convergence accuracy and speed with lower computation cost.

## 1. Introduction

Particle swarm optimization (PSO) [1] is a population-based intelligent algorithm, and it has been widely employed to solve various kinds of numerical and combinational optimization problems because of its simplicity, fast convergence, and high performance.

Researchers have proposed various modified versions of PSO to improve its performance; however, there still are premature or lower convergence rate problems. In the PSO research, how to increase population diversity to enhance the precision of solutions and how to speed up convergence rate with least computation cost are two vital issues. Generally speaking, there are four strategies to fulfill these targets as follows.

(1) Tuning control parameters. As for inertial weight, linearly decreasing inertial weight [2], fuzzy adaptive inertial weight [3], rand inertial weight [4], and adaptive inertial weight based on velocity information [5], they can enhance the performance of PSO. Concerning acceleration coefficients, the time-varying acceleration coefficients [6] are widely used. Clerc and Kennedy analyzed the convergence behavior by introducing constriction factor [7], which is proved to be equivalent to the inertial weight [8].

(2) Hybrid PSO, which hybridizes other heuristic operators to increase population diversity. The genetic operators have been hybridized with PSO, such as selection operator [9], crossover operator [10], and mutation operator [11]. Similarly, differential evolution algorithm [12], ant colony optimization [13], and local search strategy [14] have been introduced into PSO.

(3) Changing the topological structure. The global and local versions of PSO are the main type of swarm topologies. The global version converges fast with the disadvantage of trapping in local optima, while the local version can obtain a better solution with slower convergence [15]. The Von Neumann topology is helpful for solving multimodal problems and may perform better than other topologies including the global version [16].

(4) Eliminating the velocity formula. Kennedy proposed the bare-bones PSO (BPSO) [17] and variants of BPSO [18, 19]. Sun et al. proposed quantum-behaved PSO (QPSO) and relative convergence analysis [20, 21].

In recent years, some modified PSO have extremely enhanced the performance of PSO. For example, Zhan et al. proposed adaptive PSO (APSO) [22] and Wang et al. proposed so-called diversity enhanced particle swarm optimization with neighborhood search (DNSPSO) [23]. The former introduces an evolutionary state estimation (ESE) technique to adaptively adjust the inertia weight and acceleration coefficients. The later ones, a diversity enhancing mechanism and neighborhood-based search strategies, were employed to carry out a tradeoff between exploration and exploitation.

Though all kinds of variants of PSO have enhanced performance of PSO, there are still some problems such as hardly implement, new parameters to just, or high computation cost. So it is necessary to investigate how to trade off the exploration and exploitation ability of PSO and reduce the parameters sensitivity of the solved problems and improve the convergence accuracy and speed with the least computation cost and easy implementation. In order to carry out the targets, in this paper, the global worst position (solution) was introduced into the velocity equation of the standard PSO (SPSO), which is called impelled/penalized learning according to the corresponding weight coefficient. Meanwhile, we eliminate the two acceleration coefficients *c*_1_ and *c*_2_ from the SPSO to reduce the parameters sensitivity of the solved problems. The so-called HPSO has been employed to some nonlinear benchmark functions, which compose unimodal, multimodal, rotated, and shifted high-dimensional functions, to confirm its high performance by comparing with other well-known modified PSO.

The remainder of the paper is structured as follows. In Section 2, the standard particle swarm optimization (SPSO) is introduced. The proposed HPSO is given in Section 3. Experimental studies and discussion are provided in Section 4. Some conclusions are given in Section 5.

## 2. Standard PSO (SPSO)

The PSO is inspired by the behavior of bird flying or fish schooling; it is firstly introduced by Kennedy and Eberhart in 1995 [1] as a new heuristic algorithm. In the standard PSO (SPSO) [2], a swarm consists of a set of particles, and each particle represents a potential solution of an optimization problem. Considering the *i*th particle of the swarm with *N* particles in a *D*-dimensional space, its position and velocity at iteration *t* are denoted by *X*_*i*_(*t*) = (*x*_*i*_^1^(*t*), *x*_*i*_^2^(*t*),…, *x*_*i*_^*D*^(*t*)) and *V*_*i*_(*t*) = (*v*_*i*_^1^(*t*), *v*_*i*_^2^(*t*),…, *v*_*i*_^*D*^(*t*)). Then, the new velocity and position on the *d*-dimension of this particle at iteration *t* + 1 will be calculated by using the following:

(1) vid(t+1)=w·vid(t)+c1·r1d(t)·(Pbestid(t)−xid(t))+c2·r2d(t)·(Gbestd(t)−xid(t)),

where *i* = 1,2,…, *N*, and *N* is the population size; *d* = 1,2,…, *D*, and *D* is the dimension of search space; *r*_1_^*d*^ and *r*_2_^*d*^ are two uniformly distributed random numbers in the interval [0,1]; acceleration coefficients *c*_1_ and *c*_2_ are nonnegative constants which control the influence of the cognitive and social components during the search process. *P*best_*i*_(*t*) = (*P*best_*i*_^1^(*t*),…, *P*best_*i*_^*D*^(*t*)), called the personal best solution, represents the best solution found by the *i*th particle itself until iteration *t*; *G*best(*t*) = (*G*best^1^(*t*),…, *G*best^*D*^(*t*)), called the global best solution, represents the global best solution found by all particles until iteration *t*. *w* is the inertial weight to balance the global and local search abilities of particles in the search space, which is given by

(2) $$\begin{array}{c} w=w_{\max}-\frac{w_{\max}-w_{\min}}{T}\times t, \end{array}$$

where *w*_max⁡_ is the initial weight, *w*_min⁡_ is the final weight, *t* is the current iteration number, and *T* is the maximum iteration number. Then, update particle's position using the following:

(3) $$\begin{array}{c} x_{i}^{d}\left(t+1\right)=x_{i}^{d}\left(t\right)+v_{i}^{d}\left(t+1\right) \end{array}$$

and check *x*_min⁡_^*d*^ ≤ *x*_*i*_^*d*^(*t* + 1) ≤ *x*_max⁡_^*d*^, where *x*_min⁡_^*d*^ and *x*_max⁡_^*d*^ represent lower and upper bounds of the *d*th variable, respectively.

## 3. Human Behavior-Based PSO (HPSO)

In this section, a modified version of SPSO based on human behavior, which is called HPSO, is proposed to improve the performance of SPSO. In SPSO, all particles only learn from the best particles *P*best and *G*best. Obviously, it is an ideal social condition. However, considering the human behavior, there exist some people who have bad habits or behaviors around us, at the same time, as we all known that these bad habits or behaviors will bring some effects on people around them. If we take warning from these bad habits or behaviors, it is beneficial to us. Conversely, if we learn from these bad habits or behaviors, it is harmful to us. Therefore, we must give an objective and rational view on these bad habits or behavior.

In HPSO, we introduce the global worst particle, who is of the worst fitness in the entire population at each iteration. It is denoted as *G*worst and defined as follows:

(4) Gworst(t) =argmax{f(Pbest1),f(Pbest2),…,f(PbestN)},

where *f*(·) represents the fitness value of the corresponding particle.

To simulate human behavior and make full use of the *G*worst, we introduce a learning coefficient *r*_3_, which is a random number obeying the standard normal distribution; that is, *r*_3_ ∈ *N*(0,1). If *r*_3_ > 0, we consider it as an impelled learning coefficient, which is helpful to enhance the “flying” velocity of the particle; therefore, it can enhance the exploration ability of particle. Conversely, if *r*_3_ < 0, we consider it as a penalized learning coefficient, which can decrease the “flying” velocity of the particle; therefore, it is beneficial to enhance the exploitation. If *r*_3_ = 0, it represents that these bad habits or behaviors have not effect on the particle. Meanwhile, in order to reduce the parameters sensitivity of the solved problems, we take place of the two acceleration coefficients *c*_1_ and *c*_2_ with two random learning coefficients *r*_1_ and *r*_2_, respectively. Therefore, the velocity equation has been changed as follows:

(5) vid(t+1)=w·vid(t)+r1(t)·(Pbestid(t)−xid(t))+r2(t)·(Gbestd(t)−xid(t))+r3(t)·(Gworstd(t)−xid(t)),

where *r*_1_ and *r*_2_ are two random numbers in range of [0,1] and *r*_1_ + *r*_2_ = 1. The random numbers *r*_1_, *r*_2_, and *r*_3_ are the same for all *d* = 1,2,…, *D* but different for each particle, and they are generated anew in each iteration. If *v*_*i*_^*d*^(*t* + 1) overflows the boundary, we set boundary value to it. Consider

(6) $$\begin{array}{c} v_{i}^{d}\left(t+1\right)=\{\begin{array}{cc} v_{\min}^{d}, & \text{if}\;\;v_{i}^{d}\left(t+1\right)<v_{\min}^{d},\\ v_{\max}^{d}, & \text{if}\;\;v_{i}^{d}\left(t+1\right)>v_{\max}^{d},\\ v_{i}^{d}\left(t+1\right), & \text{otherwise,} \end{array} \end{array}$$

where *v*_min⁡_^*d*^ and *v*_max⁡_^*d*^ are the minimum and maximum velocity of the *d*-dimensional search space, respectively. Similarly, if *x*_*i*_^*d*^(*t* + 1) flies out of the search space, we limit it to the corresponding bound value.

**Figure 1:**
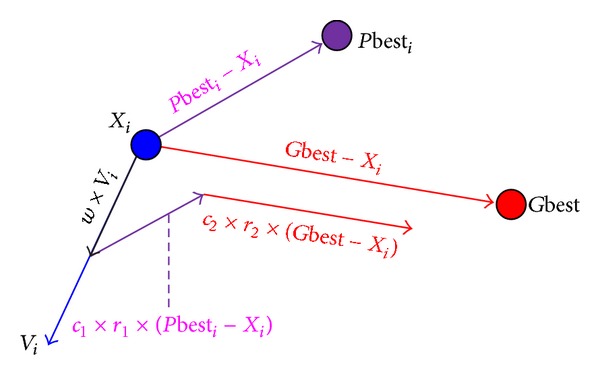
Cognition and social terms in PSO.

**Figure 2:**
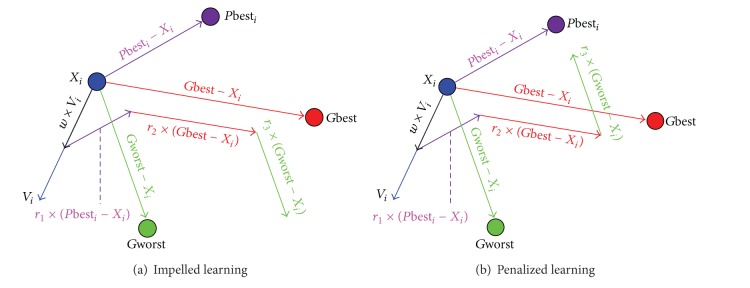
Impelled/penalized term in HPSO.

In SPSO, the cognition and social learning terms move particle *i* towards good solutions based on *P*best_*i*_ and *G*best in the search space as shown in Figure 1. This strategy makes a particle fly fast to good solutions, so it is easy to trap in local optima. From Figure 2, we can clearly observe that both impelled learning term and penalized term provide a particle with the chance to change flying direction. Therefore, the impelled/penalized term plays a key role in increasing the population diversity, which is beneficial in helping particles to escape from the local optima and enhance the convergence speed. In HPSO, the impelled/penalized learning term performs a proper tradeoff between the exploration and exploitation.

**Figure 3:**
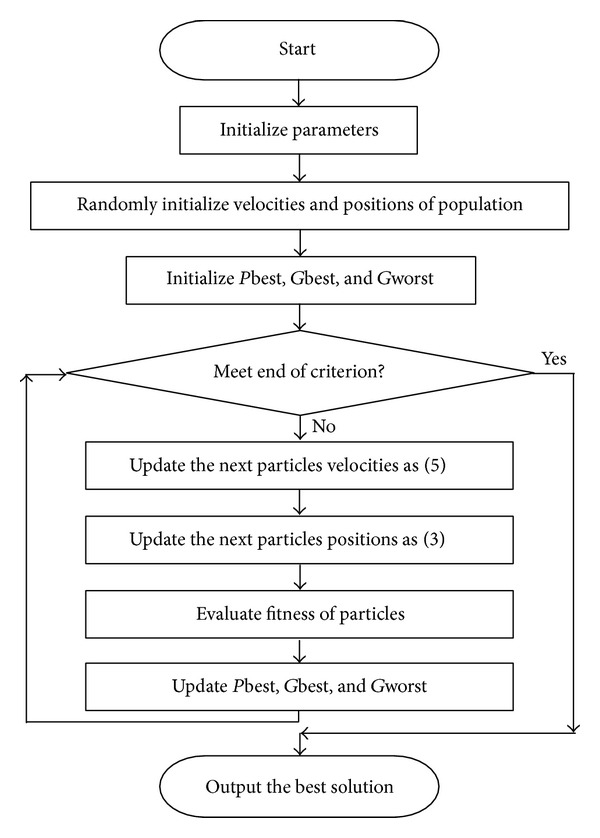
HPSO flowchart.

To sum up, Figure 3 illustrates the flowchart of HPSO. Meanwhile, the pseudocodes of implementing the HPSO are listed as shown in Algorithm 1.

## 4. Experimental Studies and Discussion

To evaluate the performance of HPSO, 28 minimization benchmark functions are selected [22, 24, 25] as detailed in Section 4.1. HPSO is compared with SPSO in different search spaces and the results are given in Section 4.2. In addition, HPSO is compared with some well-known variants of PSO in Section 4.3.

### 4.1. Benchmark Functions

In the experimental study, we choose 28 minimization benchmark functions, which consist of unimodal, multimodal, rotated, shifted, and shifted rotated functions.  Table 1 lists the main information; please refer to papers [22, 24, 25] to obtain further detailed information about these functions. Among these functions, *F*_1_–*F*_6_ are unimodal functions. *F*_7_ is the Rosenbrock function, which is unimodal for *D* = 2 and *D* = 3 but may have multiple minima in high dimension cases. *F*_8_–*F*_15_ are unrotated multimodal functions and the number of their local minima increases exponentially with the problem dimension. *F*_16_–*F*_23_ are rotated functions. *F*_24_–*F*_26_ are shifted functions and *F*_27_ and *F*_28_ are shifted rotated multimodal functions and *O* = (*o* ^1^, *o* ^2^,…, *o* ^*D*^) is a randomly generated shift vector located in the search space. To obtain a rotated function, an orthogonal matrix *M* [26] is considered and the rotated variable *y* = *M* × *x* is computed. Then, the vector *y* is used to evaluate the objective function value.

### 4.2. Comparison of HPSO with SPSO

The performance on the convergence accuracy of HPSO is compared with that of SPSO. The test functions listed in Table 1 are evaluated. For a fair comparison, we set the same parameters value. Population size is set to 30 (*N* = 30), upper bounds of velocity *V*_max⁡_ = 0.5 × (*X*_max⁡_ − *X*_min⁡_), and the corresponding lower bounds *V*_min⁡_ = −*V*_max⁡_, where *X*_min⁡_ and *X*_max⁡_ are the lower and upper bounds of variables, respectively. Inertia weight *w* is linearly decreased from 0.9 to 0.4 in SPSO and HPSO. Acceleration coefficients *c*_1_ and *c*_2_ in SPSO are set to 2. The two algorithms are independently run 30 times on the benchmark functions. The results in terms of the best, worst, median, mean, and standard deviation (SD) of the solutions obtained in the 30 independent runs by each algorithm in different search spaces are as shown in Tables 2, 3, and 4. At the same time, the maximum iteration *T* is 1000 for *D* = 30, 2000 for *D* = 50, and 3000 for *D* = 100, respectively.

From Tables 2–4, we can clearly observe that the convergence accuracy of HPSO is better than SPSO on the most benchmark functions. An interesting result is that HPSO can find the global optimal solutions on functions *F*_2_, *F*_4_, *F*_5_, *F*_9_, *F*_12_, *F*_13_, *F*_15_, *F*_19_, and *f*_22_ in all search spaces; that is to say, HPSO can obtain the 100% success rate on the listed functions. Considering *F*_1_ and *F*_10_, though HPSO can find the global optimal solutions in all different search ranges, it only obtained the mean values 333.3333 and 0.8333, respectively, in 100-dimensional space. At the same time, HPSO offers the higher convergence accuracy on functions *F*_3_, *F*_6_, *F*_7_, *F*_11_, *F*_14_, *F*_16_, *F*_20_, *F*_21_, *F*_23_, and *F*_26_. However, we must observe that SPSO has higher performance on function *F*_8_. As for *F*_25_, SPSO has better performance in 30-dimensional search space, but HPSO has better performance in 50- and 100-dimensional search spaces. As for shifted rotated functions *F*_27_ and *F*_28_, both SPSO and HPSO have worst convergence accuracy. As seen, the dimension of the selected functions has great effect on SPSO. For example, considering function *F*_1_, SPSO has mean value 666.6686, 3.6667*e* + 03, and 4.0698*e* + 04 in 30-dimensional, 50-dimensional, and 100-dimensional search spaces, respectively, while HPSO has mean values 0, 0, and 333.333 in the corresponding search space. Therefore, we also conclude that HPSO has better stability than SPSO from the data in different search spaces.

In the 9th columns of Tables 2–4, we report the statistical significance level of the difference of the means of the two algorithms. Note that here “+” indicates that the *t* value is significant at a 0.05 level of significance by two-tailed test, and “−” stands for the difference of means that is not statistically significant.

**Figure 4:**
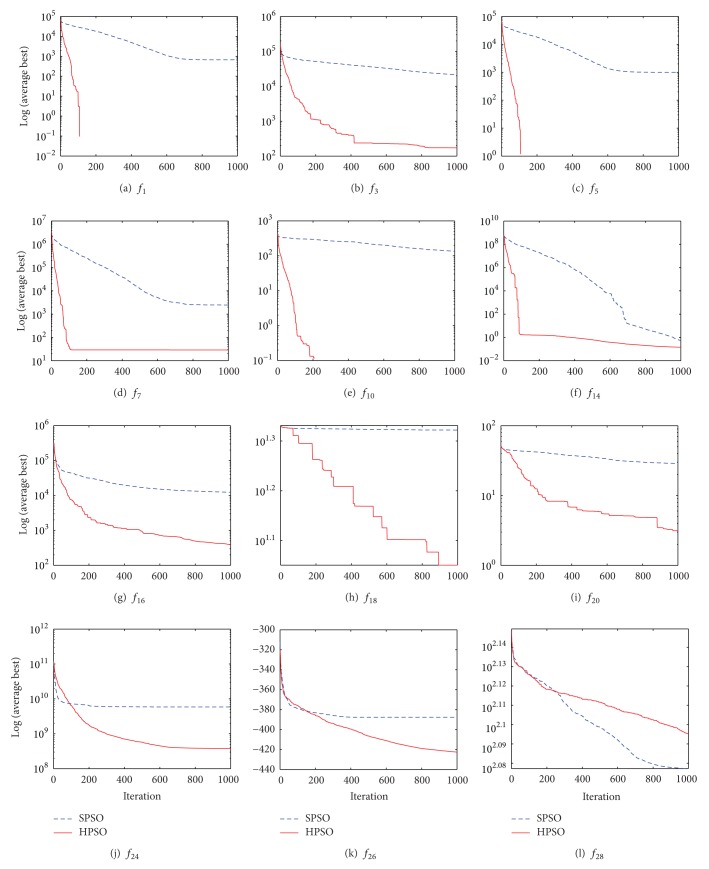
Convergence comparison of HPSO and SPSO on the selected test functions with *D* = 30, *N* = 30, and *T* = 1000.

Figure 4 graphically presents the comparison in terms of convergence characteristics of the evolutionary processes in solving the selected benchmark functions in 30-dimensional search space with *N* = 30 and *T* = 1000.

### 4.3. Comparison of HPSO with Other PSO Algorithms

In this section, a comparison of HPSO with some well-known PSO algorithms which are listed in Table 5 is performed to evaluate the efficiency of the proposed algorithm.

At first, we choose 10 unimodal and multimodal test functions for this evaluation. According to [22], the algorithms GPSO [2], LPSO [16], VPSO [27], FIPS [28], HPSO-TVAC [6], DMS-PSO [29], CLPSO [24], and APSO [22] are considered as detailed in Table 5. The experimental results of the algorithms are directly from [22] as shown in Table 6. In this trial, the population size *N* = 20, the dimension *D* = 30, and the maximum fitness evaluations (FEs) were set to 2 × 10^5^ also. The parameter configurations of the selected algorithms have been set according to their corresponding references. The inertia weight *w* is linearly decreased from 0.9 to 0.4 in HPSO. HPSO is independently run 30 times and the mean and SD are shown in Table 6. As seen, HPSO has the first rank among the algorithms and obtains the global minimum on functions *F*_1_, *F*_2_, *F*_5_, *F*_9_, *F*_10_, and *F*_12_ and gives the good near-global optima on functions *F*_6_ and *F*_11_. Meanwhile, HPSO has the worst performance on functions *F*_3_ and *F*_14_. As for *F*_3_, APSO has the best convergence accuracy, and HPSO only wins CLPSO. Considering *F*_14_, DMS-PSO has the best performance.

Then, in the next step, we choose six functions from [25] and seven algorithms of GPSO, QIPSO [30], UPSO [31], FIPS, AFSO [25], and AFSO-Q1 [25] as detailed in Table 5. For a fair comparison, the population size *N* = 30, the dimension *D* = 30, and the maximum iteration *T* = 10,000 also in HPSO, and the inertia weight *w* is linearly decreased from 0.9 to 0.4. HPSO is independently run 30 times and the mean and SD are shown in Table 7. As seen, HPSO shows better performance and has the first rank. HPSO finds the global optimal solution on functions *F*_9_, *F*_13_, *F*_21_, and *F*_22_. FIPS and UPSO have better convergence accuracy on functions *F*_27_ and *F*_28_, respectively.

Therefore, it is worth saying that the proposed algorithm has considerably better performance than the other well-known PSO algorithms in unimodal and multimodal high-dimensional functions.

## 5. Conclusion

In this paper, a modified version of PSO called HPSO has been introduced to enhance the performance of SPSO. To simulate the human behavior, the global worst particle was introduced into the velocity equation of SPSO, and the learning coefficient which obeys the standard normal distribution can balance the exploration and exploitation abilities by changing the flying direction of particles. When the coefficient is positive, it is called impelled leaning coefficient, which is helpful to enhance the exploration ability. When the coefficient is negative, it is called penalized learning coefficient, which is beneficial for improving the exploitation ability. At the same time, the acceleration coefficients *c*_1_ and *c*_2_ have been replaced with two random numbers, whose sum is equal to  1 in [0,1]; this strategy decreases the dependence on parameters of the solved problems. The proposed algorithm has been evaluated on 28 benchmark functions including unimodal, unrotated multimodal, rotated, shifted, and shifted rotated functions, and the experimental results confirm the high performance of HPSO on the main functions. However, as seen, HPSO has the worst performance on shifted rotated functions, so it is worth researching how to enhance the performance of HPSO on shifted rotated functions in the future. Meanwhile, applying HPSO to solve real-world problems is also a research field.

## Figures and Tables

**Algorithm 1:**
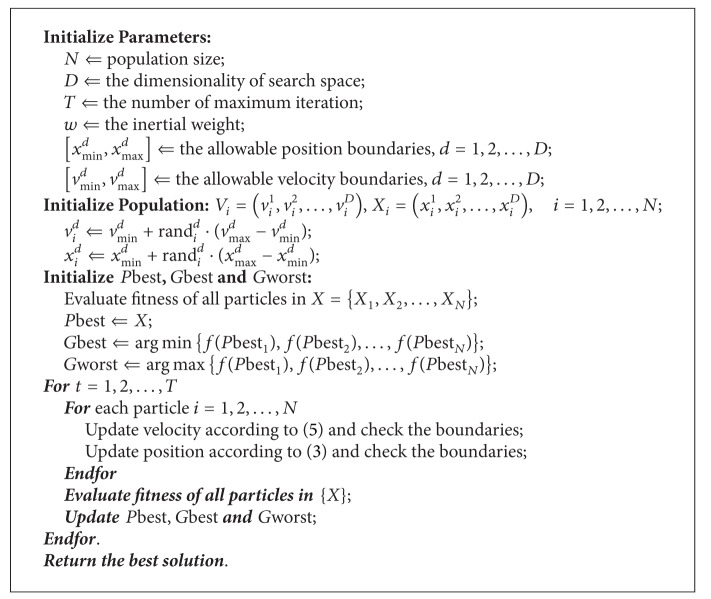
HPSO.

**Table 1:** Functions' names, dimensions, ranges, and global optimum values of benchmark functions used in the experiments.

Number	Function name	Dimension (*D*)	[Range]^*D*^	*F*_opt_
*F*_1_	Sphere model	30/50/100	[−100, 100]^D^	0
*F*_2_	Schwefel's problem 2.22	30/50/100	[−10, 10]^D^	0
*F*_3_	Schwefel's problem 1.2	30/50/100	[−100, 100]^D^	0
*F*_4_	Schwefel's problem 2.21	30/50/100	[−100, 100]^D^	0
*F*_5_	Step function	30/50/100	[−100, 100]^D^	0
*F*_6_	Quartic function, that is, noise	30/50/100	[−1.28, 1.28]^D^	0
*F*_7_	Rosenbrock's function	30/50/100	[−10, 10]^D^	0
*F*_8_	Schwefel's function	30/50/100	[−500, 500]^D^	0
*F*_9_	Generalized Rastrigin's function	30/50/100	[−5.12, 5.12]^D^	0
*F*_10_	Noncontinuous Rastrigin's function	30/50/100	[−5.12, 5.12]^D^	0
*F*_11_	Ackley's function	30/50/100	[−32, 32]^D^	0
*F*_12_	Generalized Griewank's function	30/50/100	[−600, 600]^D^	0
*F*_13_	Weierstrass's function	30/50/100	[−0.5, 0.5]^D^	0
*F*_14_	Generalized penalized function	30/50/100	[−50, 50]^D^	0
*F*_15_	Cosine mixture problem	30/50/100	[−1, 1]^D^	−0.1 × *D*
*F*_16_	Rotated elliptic function	30/50/100	[−1.28, 1.28]^D^	0
*F*_17_	Rotated Schwefel's function	30/50/100	[−500, 500]^D^	0
*F*_18_	Rotated Ackley's function	30/50/100	[−32, 32]^D^	0
*F*_19_	Rotated Griewank's function	30/50/100	[−600, 600]^D^	0
*F*_20_	Rotated Weierstrass's function	30/50/100	[−0.5, 0.5]^D^	0
*F*_21_	Rotated Rastrigin's function	30/50/100	[−5.12, 5.12]^D^	0
*F*_22_	Rotated Salomon's function	30/50/100	[−100, 100]^D^	0
*F*_23_	Rotated Rosenbrock's function	30/50/100	[−100, 100]^D^	0
*F*_24_	Shifted Rosenbrock's function	30/50/100	[−100, 100]^D^	390
*F*_25_	Shifted Rastrigin's function	30/50/100	[−5, 5]^D^	−330
*F*_26_	Shifted Schwefel's problem 2.21	30/50/100	[−100, 100]^D^	−450
*F*_27_	Shifted rotated Ackley's function	30/50/100	[−32, 32]^D^	−140
*F*_28_	Shifted rotated Weierstrass's function	30/50/100	[−0.5, 0.5]^D^	90

**Table 2:** Experimental results obtained by SPSO and HPSO on function from *F*_1_ to *F*_10_.

Fun	Dim		Best	Worst	Meadian	Mean	SD	Significant
*F*_1_	30	SPSO	1.1992*e* − 04	1.0000*e* + 04	9.9690*e* − 04	666.6686	2.5371*e* + 03	
		HPSO	**0 **	**0 **	**0 **	**0 **	**0 **	**+**
	50	SPSO	9.4288*e* − 04	1.0000*e* + 04	0.0078	3.6667*e* + 03	3.6667*e* + 03	
		HPSO	**0 **	**0 **	**0 **	**0 **	**0 **	**+**
	100	SPSO	1.0013*e* + 04	7.0017*e* + 04	4.0087*e* + 04	4.0698*e* + 04	2.0974*e* + 04	
		HPSO	**0 **	**10000 **	**0 **	**333.3333 **	1.8257**e** + 03	**+**

*F*_2_	30	SPSO	6.8555*e* − 04	30.0018	10.0017	11.3364	10.0777	
		HPSO	**0 **	**0 **	**0 **	**0 **	**0 **	**+**
	50	SPSO	0.0329	70.0010	40.0006	37.3438	15.2918	
		HPSO	**0 **	**0 **	**0 **	**0 **	**0 **	**+**
	100	SPSO	51.0214	181.4054	110.5934	114.3039	29.0723	
		HPSO	**0 **	**0 **	**0 **	**0 **	**0 **	**+**

*F*_3_	30	SPSO	6.4613*e* + 03	3.7311*e* + 04	2.2333*e* + 04	2.1337*e* + 04	6.7035*e* + 03	
		HPSO	**0 **	5.1779**e** + 03	**0**	**172.5975 **	**945.3557 **	**+**
	50	SPSO	4.0023*e* + 04	1.0191*e* + 05	6.5660*e* + 04	7.0328*e* + 04	1.7603*e* + 04	
		HPSO	**0 **	6.9787**e** + 03	**0**	**232.6222 **	1.2741**e** + 03	**+**
	100	SPSO	1.7694*e* + 05	3.0086*e* + 05	2.4789*e* + 05	2.4752*e* + 05	3.6623*e* + 04	
		HPSO	**0 **	2.6987**e** + 04	**0 **	3.8008**e** + 03	6.9150**e** + 03	**+**

*F*_4_	30	SPSO	8.6091	21.2711	12.9945	13.3502	3.5341	
		HPSO	**0 **	**0 **	**0 **	**0 **	**0 **	**+**
	50	SPSO	24.2031	39.5127	31.0562	31.1715	4.2886	
		HPSO	**0 **	**0 **	**0 **	**0 **	**0 **	**+**
	100	SPSO	54.1172	75.3686	64.7834	64.2358	4.2202	
		HPSO	**0 **	**0 **	**0 **	**0 **	**0 **	**+**

*F*_5_	30	SPSO	**0 **	10001	**0 **	1.0005*e* + 03	3.0512*e* + 03	
		HPSO	**0 **	**0 **	**0 **	**0 **	**0 **	**+**
	50	SPSO	**0 **	20004	4.5000	5.0028*e* + 03	6.8230*e* + 03	
		HPSO	**0 **	**0 **	**0 **	**0 **	**0 **	**+**
	100	SPSO	127	90040	40068	4.3086*e* + 04	2.2747*e* + 04	
		HPSO	**0 **	**0 **	**0 **	**0 **	**0 **	**+**

*F*_6_	30	SPSO	0.0344	18.8556	0.0959	3.5587	5.1400	
		HPSO	1.4522**e** − 04	**0.0030 **	**0.0012 **	**0.0012 **	8.5738**e** − 04	**+**
	50	SPSO	0.0780	72.6594	13.6489	19.6604	19.3860	
		HPSO	7.4623**e** − 05	**0.0017 **	5.3645**e** − 04	6.3534**e** − 04	4.7283**e** − 04	**+**
	100	SPSO	86.7855	381.9209	200.8146	211.9720	88.3159	
		HPSO	3.5210**e** − 05	**0.0019 **	2.9387**e** − 04	4.0826**e** − 04	3.5395**e** − 04	**+**

*F*_7_	30	SPSO	**14.3237 **	1.0083*e* + 04	140.5176	2.4686*e* + 03	4.2581*e* + 03	
		HPSO	28.6353	**28.9456 **	**28.8793 **	**28.8461 **	**0.0932 **	**+**
	50	SPSO	97.0317	9.4285*e* + 05	376.2306	3.4093*e* + 04	1.7169*e* + 05	
		HPSO	**48.4886 **	**48.8766 **	**48.7600 **	**48.7513 **	**0.0875 **	**+**
	100	SPSO	706.1328	2.8333*e* + 06	9.4375*e* + 05	8.8851*e* + 05	8.9157*e* + 05	
		HPSO	**98.4280 **	**98.8373 **	**98.7133 **	**98.7129 **	**0.0818 **	**+**

*F*_8_	30	SPSO	2.0226**e** + 03	4.8935**e** + 03	3.5787**e** + 03	3.6128**e** + 03	**733.1063 **	
		HPSO	3.5886*e* + 03	8.0516*e* + 03	6.6047*e* + 03	6.3505*e* + 03	1.0893*e* + 03	−
	50	SPSO	5.8499**e** + 03	9.7913**e** + 03	7.8862**e** + 03	7.7139**e** + 03	1.0101**e** + 03	
		HPSO	6.5496*e* + 03	1.4460*e* + 04	1.1191*e* + 04	1.0866*e* + 04	2.1757*e* + 03	−
	100	SPSO	1.8110*e* + 04	2.4259**e** + 04	2.0949**e** + 04	2.1084**e** + 04	1.7384**e** + 03	
		HPSO	1.2615**e** + 04	3.1402*e* + 04	2.4302*e* + 04	2.4077*e* + 04	4.9510*e* + 03	**−**

*F*_9_	30	SPSO	28.7299	160.3815	87.6754	92.5142	32.6994	
		HPSO	**0 **	**0 **	**0 **	**0 **	**0 **	**+**
	50	SPSO	175.2643	351.6480	260.4359	258.0518	48.4078	
		HPSO	**0 **	**0 **	**0 **	**0 **	**0 **	**+**
	100	SPSO	555.8950	993.3887	750.1694	749.1658	749.1658	
		HPSO	**0 **	**0 **	**0 **	**0 **	**0 **	**+**

*F*_10_	30	SPSO	61.4129	221.0445	132.7694	134.5414	33.8073	
		HPSO	**0 **	**0 **	**0 **	**0 **	**0 **	**+**
	50	SPSO	157.1020	440.0897	324.2632	310.3595	64.3675	
		HPSO	**0 **	**0 **	**0 **	**0 **	**0 **	**+**
	100	SPSO	623.5658	1.0433*e* + 03	804.6981	813.3435	88.5932	
		HPSO	**0 **	**25 **	**0 **	**0.8333**	**4.5644 **	**+**

**Table 3:** Experimental results obtained by SPSO and HPSO on functions from *F*_11_ to *F*_20_.

Fun	Dim		Best	Worst	Median	Mean	SD	Significant
*F*_11_	30	SPSO	0.0043	19.9630	0.0595	2.3935	5.4041	
		HPSO	8.8818**e** − 16	8.8818**e** − 16	8.8818**e** − 16	8.8818**e** − 16	**0 **	**+**
	50	SPSO	0.0598	19.9646	12.6912	10.5673	6.3042	
		HPSO	8.8818**e** − 16	8.8818**e** − 16	8.8818**e** − 16	8.8818**e** − 16	**0 **	**+**
	100	SPSO	15.4237	20.2143	19.5200	19.4135	0.8672	
		HPSO	8.8818**e** − 16	8.8818**e** − 16	8.8818**e** − 16	8.8818**e** − 16	**0 **	**+**

*F*_12_	30	SPSO	7.0274*e* − 04	90.8935	0.0178	12.0794	31.2763	
		HPSO	**0 **	**0 **	**0 **	**0 **	**0 **	**+**
	50	SPSO	0.0014	270.8170	0.0415	45.1971	70.1274	
		HPSO	**0 **	**0 **	**0 **	**0 **	**0 **	**+**
	100	SPSO	1.1140	721.0594	361.0858	376.1758	158.6584	
		HPSO	**0 **	**0 **	**0 **	**0 **	**0 **	**+**

*F*_13_	30	SPSO	0.1403	4.3952	0.3210	1.0567	1.4863	
		HPSO	**0 **	**0 **	**0 **	**0 **	**0 **	**+**
	50	SPSO	0.8657	15.2389	7.5828	8.2388	3.6607	
		HPSO	**0 **	**0 **	**0 **	**0 **	**0 **	**+**
	100	SPSO	27.6235	64.4826	49.3984	47.7138	10.0126	
		HPSO	**0 **	**0 **	**0 **	**0 **	**0 **	**+**

*F*_14_	30	SPSO	6.4114*e* − 05	2.2031	0.4202	0.5373	0.5730	
		HPSO	0.0710	0.2803	0.1301	0.1444	0.0513	+
	50	SPSO	0.1882	6.9784	2.2774	2.3889	1.5688	
		HPSO	0.1016	0.3137	0.1652	0.1702	0.0438	+
	100	SPSO	32.5063	5.1200*e* + 08	457.9143	7.6801*e* + 07	1.5257*e* + 08	
		HPSO	0.1866	0.5097	0.2703	0.2736	0.0653	+

*F*_15_	30	SPSO	−3.0000	−2.8522	−3.0000	−2.9507	0.0709	
		HPSO	**−3 **	**−3 **	**−3 **	**−3 **	**0 **	**+**
	50	SPSO	−5.0000	−2.3044	−4.4827	−4.2127	0.6865	
		HPSO	**−5 **	**−5 **	**−5 **	**−5 **	**0 **	**+**
	100	SPSO	−7.9165	4.7637	−5.2127	−4.6977	2.8465	
		HPSO	**−10 **	**−10 **	**−10 **	**−10 **	**0 **	**+**

*F*_16_	30	SPSO	2.3604*e* + 03	3.8233*e* + 04	3.8233*e* + 04	1.2375*e* + 04	9.2463*e* + 03	
		HPSO	0	5.8369*e* + 03	0	390.6710	1.2756*e* + 03	+
	50	SPSO	7.1213*e* + 03	1.0427*e* + 05	3.3195*e* + 04	3.4891*e* + 04	2.2914*e* + 04	
		HPSO	0	4.0529*e* + 03	0	224.6749	873.6249	+
	100	SPSO	6.2317*e* + 04	2.7386*e* + 05	1.4222*e* + 05	1.4697*e* + 05	5.7699*e* + 04	
		HPSO	0	1.9403*e* + 04	0	1.0583*e* + 03	3.8088*e* + 03	+

*F*_17_	30	SPSO	6.7986*e* + 03	9.7587*e* + 03	8.3387*e* + 03	8.2508*e* + 03	739.7223	
		HPSO	8.3590*e* + 03	9.8803*e* + 03	9.0866*e* + 03	9.0790*e* + 03	442.4330	−
	50	SPSO	1.3020*e* + 04	1.7080*e* + 04	1.4999*e* + 04	1.5149*e* + 04	1.0581*e* + 03	
		HPSO	1.5003*e* + 04	1.7349*e* + 04	1.6514*e* + 04	1.6310*e* + 04	669.3538	−
	100	SPSO	2.7400*e* + 04	2.7400*e* + 04	3.1087*e* + 04	3.1149*e* + 04	2.1654*e* + 03	
		HPSO	3.0329*e* + 04	3.5493*e* + 04	3.4226*e* + 04	3.3586*e* + 04	1.5320*e* + 03	−

*F*_18_	30	SPSO	20.7888	21.0951	21.0053	20.9848	0.0712	
		HPSO	8.8818*e* − 16	21.1210	20.9931	11.2354	10.6894	+
	50	SPSO	21.0515	21.2478	21.1455	21.1436	0.0536	
		HPSO	8.8818*e* − 16	21.2404	21.1366	12.0016	10.6745	+
	100	SPSO	21.2367	21.3931	21.3368	21.3358	0.0364	
		HPSO	8.8818*e* − 16	21.3949	21.3545	15.6658	9.6084	+

*F*_19_	30	SPSO	1.0517	495.3131	273.6408	243.6176	154.3551	
		HPSO	**0 **	**0 **	**0 **	**0 **	**0 **	**+**
	50	SPSO	265.0558	1.4393*e* + 03	798.8065	786.0782	289.8401	
		HPSO	**0 **	**0 **	**0 **	**0 **	**0 **	**+**
	100	SPSO	1.9937*e* + 03	4.0158*e* + 03	2.9388*e* + 03	2.9263*e* + 03	543.9053	
		HPSO	**0 **	**0 **	**0 **	**0 **	**0 **	**+**

*F*_20_	30	SPSO	22.5705	34.8494	28.6842	28.8734	3.5028	
		HPSO	0	39.9834	0	3.1393	9.7817	+
	50	SPSO	45.9462	70.7399	55.5532	55.6014	5.7839	
		HPSO	0	66.4051	0	2.2135	12.1239	+
	100	SPSO	106.4483	139.8394	120.6118	121.4481	7.8030	
		HPSO	0	129.4941	0	8.3487	31.7918	+

**Table 4:** Experimental results obtained by SPSO and HPSO on functions from *F*_21_ to *F*_28_.

Fun	Dim		Best	Worst	Median	Mean	SD	Significant
*F*_21_	30	SPSO	67.1541	307.3070	213.8939	203.8842	61.8125	
		HPSO	**0 **	**0 **	**0 **	**0 **	**0 **	**+**
	50	SPSO	158.2955	715.0245	518.1705	500.5593	135.5998	
		HPSO	0	269.3463	0	8.9782	49.1757	+
	100	SPSO	1.0850*e* + 03	1.9021*e* + 03	1.5793*e* + 03	1.5669*e* + 03	190.5584	
		HPSO	0	582.0882	0	35.5882	136.0270	+

*F*_22_	30	SPSO	0.7999	14.9999	1.2522	2.9025	4.3553	
		HPSO	**0 **	**0 **	**0 **	**0 **	**0 **	**+**
	50	SPSO	2.0999	26.0999	13.9628	12.8291	6.9033	
		HPSO	**0 **	**0 **	**0 **	**0 **	**0 **	**+**
	100	SPSO	16.5013	41.9999	35.4551	33.9791	6.3075	
		HPSO	**0 **	**0 **	**0 **	**0 **	**0 **	**+**

*F*_23_	30	SPSO	81.0577	4.0119*e* + 09	2.0685*e* + 08	6.8745*e* + 08	1.0469*e* + 09	
		HPSO	28.8214	28.9856	28.9323	28.9252	0.0421	+
	50	SPSO	3.7253*e* + 03	2.1495*e* + 10	3.6515*e* + 09	3.6515*e* + 09	5.3957*e* + 09	
		HPSO	48.7069	48.8900	48.8205	48.8139	0.0479	+
	100	SPSO	6.7997*e* + 09	9.2655*e* + 10	3.3160*e* + 10	3.8223*e* + 10	2.0050*e* + 10	
		HPSO	98.6590	98.8846	98.8109	98.7983	0.0545	+

*F*_24_	30	SPSO	6.2312*e* + 08	2.3418*e* + 10	4.9110*e* + 09	5.8767*e* + 09	5.6099*e* + 09	
		HPSO	5.9432*e* + 05	6.2859*e* + 09	7.6373*e* + 06	3.7982*e* + 08	1.2316*e* + 09	+
	50	SPSO	4.3540*e* + 09	3.3195*e* + 10	1.3961*e* + 10	1.6077*e* + 10	8.3270*e* + 09	
		HPSO	3.9454*e* + 06	8.9387*e* + 09	3.1766*e* + 07	7.0962*e* + 08	1.9565*e* + 09	+
	100	SPSO	4.9031*e* + 10	1.5465*e* + 11	9.1986*e* + 10	9.7151*e* + 10	2.8460*e* + 10	
		HPSO	2.0551*e* + 08	5.4553*e* + 09	6.7593*e* + 08	1.1373*e* + 09	1.2367*e* + 09	+

*F*_25_	30	SPSO	−229.5551	−78.6646	−176.9746	−174.7148	35.8633	
		HPSO	−204.3636	−100.1465	−148.1389	−149.7299	27.1636	−
	50	SPSO	−77.4305	156.8323	22.8512	24.6168	62.2086	
		HPSO	−102.9219	132.8077	−16.6107	−4.1921	58.2317	+
	100	SPSO	475.3838	860.0386	612.6947	632.8693	100.6069	
		HPSO	394.3532	805.2473	581.1779	590.3932	80.6175	+

*F*_26_	30	SPSO	−425.5452	−331.1195	−385.1191	−387.6682	22.2647	
		HPSO	−439.6877	−399.0205	−423.4928	−422.5533	11.3496	+
	50	SPSO	−399.6029	−326.6739	−379.4869	−370.8387	18.7600	
		HPSO	−415.6822	−391.7124	−401.4635	−400.8395	6.5162	+
	100	SPSO	−358.3688	−300.6930	−322.8060	−324.4641	15.5861	
		HPSO	−380.3478	−360.8031	−369.0319	−370.4683	5.1369	+

*F*_27_	30	SPSO	−119.2212	−118.8710	−119.0179	−119.0258	0.0866	
		HPSO	−119.1100	−118.8700	−118.9469	−118.9589	0.0545	−
	50	SPSO	−119.0222	−118.7656	−118.8316	−118.8535	0.0603	
		HPSO	−118.9117	−118.7327	−118.7780	−118.7911	0.0421	−
	100	SPSO	−118.7259	−118.6013	−118.6485	−118.6537	0.0310	
		HPSO	−118.6872	−118.5986	−118.6231	−118.6289	0.0204	−

*F*_28_	30	SPSO	113.2663	126.0977	118.5782	119.4693	3.6330	
		HPSO	114.4722	132.2305	124.3094	124.5205	4.3399	−
	50	SPSO	137.8303	153.5400	145.1433	145.1503	4.2018	
		HPSO	141.9493	162.4008	153.9547	153.1087	5.4273	−
	100	SPSO	194.1222	232.4306	215.9257	215.9174	8.6772	
		HPSO	212.5258	245.0126	229.4886	230.4426	7.4650	−

**Table 5:** Some well-known PSOs algorithms in the literature.

Algorithm	Year	Topology	Parameter settings
GPSO	1998	Global star	*w*: 0.9 − 0.4, *c*_1_ = *c*_2_ = 2.0
LPSO	2002	Local ring	*w*: 0.9 − 0.4, *c*_1_ = *c*_2_ = 2.0
FIPS	2004	Local Uring	χ = 0.729, ∑*c*_*i*_ = 4.1
HPSO-TVAC	2004	Global star	*w*: 0.9 − 0.4, *c*_1_ : 2.5 − 0.5, and *c*_2_: 0.5 − 2.5
UPSO	2004	Global star	*w*: 0.9 − 0.4, *c*_1_ = *c*_2_ = 2.0, and *U* = 0.5
DMS-PSO	2005	Dynamic multiswarm	*w*: 0.9 − 0.2, *c*_1_ = *c*_2_ = 2.0, *m* = 3, and *R* = 5
VPSO	2006	Local Von Neumann	*w*: 0.9 − 0.4, *c*_1_ = *c*_2_ = 2.0
CLPSO	2006	Comprehensive learning	*w*: 0.9 − 0.4, *c* = 1.49445, and *m* = 7
QIPSO	2007	Global star	*w*: 0.9 − 0.4, *c*_1_ = *c*_2_ = 2.0
APSO	2009	Global star	*w*: 0.9, *c*_1_ = *c*_2_ = 2.0; δ : random in [0.05,0.1], σ: 1 − 0.1
AFPSO	2011	Global star	*w*: 0.9 − 0.4, *c*_1_, and *c*_2_ are based on fuzzy rule
AFPSO-QI	2011	Global star	*w*: 0.9 − 0.4, *c*_1_, and *c*_2_ are based on fuzzy rule

**Table 6:** Comparison results of eight PSO algorithms [22] with HPSO on 10 functions (*N* = 20, *D* = 30, and FEs = 2 × 10^5^).

Function	GPSO	LPSO	VPSO	FIPS	HPSO-TVAC	DMS-PSO	CLPSO	APSO	HPSO
*F*_1_									
Mean	1.98*e* − 53	4.77*e* − 29	5.11*e* − 38	3.21*e* − 30	3.38*e* − 41	3.85*e* − 54	1.89*e* − 19	1.45*e* − 150	**0**
SD	7.08*e* − 53	1.13*e* − 28	1.91*e* − 37	3.60*e* − 30	8.50*e* − 41	1.75*e* − 53	1.49*e* − 19	5.73*e* − 150	**0**
Rank	4	8	6	7	5	3	9	2	**1**
*F*_2_									
Mean	2.51*e* − 34	2.03*e* − 20	6.29*e* − 27	1.32*e* − 17	6.9*e* − 23	2.61*e* − 29	1.01*e* − 13	5.15*e* − 84	**0**
SD	5.84*e* − 34	2.89*e* − 20	8.68*e* − 27	7.86*e* − 18	6.89*e* − 23	6.6*e* − 29	6.51*e* − 14	1.44*e* − 83	**0**
Rank	3	7	5	8	6	4	9	2	**1**
*F*_3_									
Mean	6.45*e* − 2	18.60	1.44	0.77	2.89*e* − 7	47.5	395	1.0**e** − 10	167
SD	9.45*e* − 2	30.71	1.55	0.86	2.97*e* − 7	56.4	142	2.13**e** − 10	913
Rank	3	6	5	4	2	7	9	**1 **	8
*F*_5_									
Mean	**0 **	**0 **	**0 **	**0 **	**0 **	**0 **	**0 **	**0 **	**0**
SD	**0 **	**0 **	**0 **	**0 **	**0 **	**0 **	**0 **	**0 **	**0**
Rank	**1 **	**1 **	**1 **	**1 **	**1 **	**1 **	**1 **	**1 **	**1**
*F*_6_									
Mean	7.77*e* − 3	1.49*e* − 2	1.08*e* − 2	2.55*e* − 3	5.54*e* − 2	1.1*e* − 2	3.92*e* − 3	4.66*e* − 3	1.03**e** − 04
SD	2.42*e* − 3	5.66*e* − 3	3.24*e* − 3	6.25*e* − 4	2.08*e* − 2	3.94*e* − 3	1.14*e* − 3	1.7*e* − 3	8.99**e** − 05
Rank	5	8	6	2	9	7	3	4	**1**
*F*_9_									
Mean	30.7	34.90	34.09	29.98	2.39	28.1	2.57*e* − 11	5.8*e* − 15	**0**
SD	8.68	7.25	8.07	10.92	3.71	6.42	6.64*e* − 11	1.01*e* − 14	**0**
Rank	7	9	8	6	4	5	3	2	**1**
*F*_10_									
Mean	15.5	30.40	21.33	35.91	1.83	32.8	0.167	4.14*e* − 16	**0**
SD	7.4	9.23	9.46	9.49	2.65	6.49	0.379	1.45*e* − 15	**0**
Rank	5	7	6	9	4	8	3	2	**1**
*F*_11_									
Mean	1.15*e* − 14	1.85*e* − 14	1.4*e* − 14	7.69*e* − 15	2.06*e* − 10	8.52*e* − 15	2.01*e* − 12	1.11*e* − 14	8.88**e** − 16
SD	2.27*e* − 15	4.80*e* − 15	3.48*e* − 15	9.33*e* − 16	9.45*e* − 10	1.79*e* − 15	9.22*e* − 13	3.55*e* − 15	**0**
Rank	5	7	6	2	9	3	8	4	**1**
*F*_12_									
Mean	2.37*e* − 2	1.10*e* − 2	1.31*e* − 2	9.04*e* − 4	1.07*e* − 2	1.31*e* − 2	6.45*e* − 13	1.67*e* − 2	**0**
SD	2.57*e* − 2	1.60*e* − 2	1.35*e* − 2	2.78*e* − 3	1.14*e* − 2	1.73*e* − 2	2.07*e* − 12	2.41*e* − 2	**0**
Rank	9	5	6	3	4	7	2	8	**1**
*F*_14_									
Mean	1.04*e* − 2	2.18*e* − 30	3.46*e* − 3	1.22*e* − 31	7.07*e* − 30	2.05**e** − 32	1.59*e* − 21	3.76*e* − 31	1.70*e* − 2
SD	3.16*e* − 2	5.14*e* − 30	1.89*e* − 2	4.85*e* − 32	4.05*e* − 30	8.12**e** − 33	1.93*e* − 21	1.2*e* − 30	1.42*e* − 2
Rank	8	4	7	2	5	**1 **	6	3	9

Average rank	5	6.2	5.6	4.4	4.9	4.6	5.3	2.9	**2.5**
Final rank	6	9	8	3	5	4	7	2	**1**

**Table 7:** Comparison results of seven PSO algorithms [25] with HPSO on six functions (*N* = 30, *D* = 30, and *T* = 10,000).

Function	SPSO	QIPSO	UPSO	FIPS	CLPSO	AFSO	AFSO-Q1	HPSO
*F*_9_								
Mean	52.30	25.61	59.40	106.1	74.39	17.93	15.69	**0**
SD	27.35	15.98	58.05	30.54	9.77	5.63	4.47	**0**
Rank	5	4	6	8	7	3	2	**1**
*F*_13_								
Mean	0.534	36.38	8.70	6.40	1.39*e* − 03	4.52*e* − 03	1.50*e* − 03	**0**
SD	1.74	4.66	3.08	3.04	3.28*e* − 04	9.20*e* − 03	3.48*e* − 03	**0**
Rank	5	8	7	6	2	4	3	**1**
*F*_21_								
Mean	320.2	317.5	309.5	434.1	263.3	266.3	253.3	**0**
SD	14.70	23.24	25.88	34.99	11.96	12.00	12.63	**0**
Rank	7	6	5	8	3	4	2	**1**
*F*_22_								
Mean	17.03	15.20	14.29	26.60	11.94	10.38	8.46	**0**
SD	2.55	1.32	2.15	1.42	1.37	1.38	0.948	**0**
Rank	7	6	5	8	4	3	2	**1**
*F*_27_								
Mean	−119.10	−119.10	−119.10	−119.90	−119.00	−119.70	−119.80	−119.05
SD	7.09*e* − 02	5.68*e* − 02	3.24*e* − 02	3.78*e* − 02	4.28*e* − 02	3.85*e* − 02	5.45*e* − 02	5.50*e* − 02
Rank	4	4	4	1	6	3	2	5
*F*_28_								
Mean	115.90	121.90	**113.20**	113.60	118.30	123.20	123.10	117.32
SD	2.90	4.90	6.14	3.63	2.40	**2.25 **	3.01	3.65
Rank	3	6	**1 **	**2 **	**5 **	8	7	4

Average rank	5.17	5.67	4.67	5.50	4.50	4.17	3.00	**2.17**
Final rank	6	8	5	7	4	3	2	**1 **
